# How to Administer Iron

**Published:** 1892-04

**Authors:** 


### HOW TO ADMINISTER IRON.

It is generally conceded that the officinal tincture of chloride of iron is the most valuable of the iron preparations therapeutically. The practical difficulties attending its administration for a length of time have been its disagreeably astringent taste, its corrosive action on the teeth, and its constipating action.

Dr. G. W. Weld's extensive experience in the practice of dentistry led him to recognize the virtues of the tincture of chloride of iron as a stimulant resource for patients after the strains of the dentist's work. Repeated experiments to obtain a formula free from the objectionable features resulted in the preparation of a highly palatable syrup with all the therapeutic efficacy preserved. This has been extensively tested and placed in the hands of Parke, Davis & Co. for manufacture, who strongly commend it to the medical profession for trial. Being prepared after Dr. Weld's formula, it is entitled Weld's Syrup of Iron Chloride (P., D. & Co.'s). It is believed it will effect a revolution in iron administration.

